# Promoting Post-Restraint Patient Debriefing in an Acute Psychiatric Inpatient setting

**DOI:** 10.1192/bjo.2024.385

**Published:** 2024-08-01

**Authors:** Harry Jackson, Munzir Quraishy, Maya Mendoza, Alex Berry

**Affiliations:** 1 Camden and Islington NHS Foundation Trust, London, United Kingdom; 2 Barnet, Enfield and Haringey Mental Health NHS Trust, London, United Kingdom; 3 National Hospital for Neurology and Neurosurgery, London, United Kingdom

## Abstract

**Aims:**

Controlled physical restraint is a commonly used, but controversial practice in inpatient psychiatric settings, at times bringing psychiatric practice into potential conflict with accepted medical ethical standards for preserving autonomy and bodily-integrity. However, physical restraint can produce high levels of patient distress, re-traumatise those who have experienced physical or sexual abuse, and may lead to inadvertent bodily injury, and even death on rare occasions. There is an international consensus to attempt to reduce restrictive practices, including physical restraint, as demonstrated in the World Health Organization's Quality Rights Initiative. Post-restraint patient debriefing can promote recovery, prevent future restraint, and promote a more ethical and humanising care environment.

We aimed to audit the frequency of restraint events, and post-restraint debriefs offered to patients in a single, London-based, male acute psychiatric ward.

**Methods:**

In the pre-intervention sample, data was extracted from the records of patients admitted over a six-month period (n = 75), to identify the number of patients who had undergone restraint and the number who had been debriefed. The search terms “restrain”, “PMVA”, “response team” and “debrief” were used. After each restraint event, the notes for the following two weeks were reviewed to see if a debrief was delivered.

The intervention consisted of a single description and dissemination of the results in a ward business meeting, with instruction that all staff members within the ward multidisciplinary team can help provide debrief if appropriate to do so. Where a patient was known to have been restrained, debriefs were offered during subsequent ward round reviews as appropriate.

In the post-intervention sample, we collected data from patients admitted over a 10-month period (n = 89).

We used Chi-Squared testing to compare categorical variables pre- and post-intervention.

**Results:**

Pre-intervention, 15 patients underwent restraint and of these, 8 patients (53.33%) were debriefed. Post-intervention, 21 patients underwent restraint and of these, 10 patients (47.62%) were debriefed. There was no statistical difference in the proportion of patients offered a psychological debrief (p = 0.735).

**Conclusion:**

Following a single intervention there was not a sustained difference in the proportion of post-restraint debriefs offered. It is likely more sustained interventions would bring about more substantive practice change. Incorporating the need for post-restraint debriefs in daily ward safety-huddles, or in structured “ward round proformas”, may increase the proportion of patients offered post-restraint debriefing. It is possible that the note review strategy did not capture all debriefs delivered.

